# Influence of Functional Group Concentration on Hypercrosslinking of Poly(vinylbenzyl chloride) PolyHIPEs: Upgrading Macroporosity with Nanoporosity

**DOI:** 10.3390/polym13162721

**Published:** 2021-08-14

**Authors:** Amadeja Koler, Mitja Kolar, Karel Jeřábek, Peter Krajnc

**Affiliations:** 1 PolyOrgLab, Faculty of Chemistry and Chemical Engineering, University of Maribor, Smetanova 17, SI-2000 Maribor, Slovenia; amadeja.koler@um.si; 2 Faculty of Chemistry and Chemical Technology, University of Ljubljana, Večna Pot 113, SI-1000 Ljubljana, Slovenia; mitja.kolar@fkkt.uni-lj.si; 3 Institute of Chemical Process Fundamentals of the Czech Academy of Sciences, Rozvojova 2/135, CZ-165 02 Prague, Czech Republic; jerabek@icpf.cas.cz

**Keywords:** polyHIPE, hypercrosslinking, poly(4-vinylbenzyl chloride), hierarchically porous polymers, nanoporosity, macroporosity

## Abstract

With the aim to study the influence of monomer ratio in poly(high internal phase emulsions) (polyHIPEs) on the polymer network architecture and morphology of poly(vinylbenzyl chloride-co-divinylbenzene-co-styrene) after hypercrosslinking via the internal Friedel–Crafts process, polyHIPEs with 80% overall porosity were prepared at three different initial crosslinking degrees, namely 2, 5, and 10 mol.%. All had typical interconnected cellular morphology, which was not affected by the hypercrosslinking process. Nitrogen adsorption and desorption experiments with BET and *t*-plot modelling were used for the evaluation of the newly introduced nanoporosity and in combination with elemental analysis for the evaluation of the extent of the hypercrosslinking. It was found that, for all three initial crosslinking degrees, the minimum amount of functional monomer, 4-vinylbenzyl chloride, was approximately 30 mol.%. Hypercrosslinking of polymers with lower concentrations of functional monomer did not result in induction of nanoporosity while the initial crosslinking degree had a much lower impact on the formation of nanoporosity.

## 1. Introduction

The concept of polymer chain hypercrosslinking was introduced by Davankov, Rogoshin and Tsyurupa, using linear polystyrene or swollen gel-type poly (styrene-co-divinylbenzene) in the presence of an external crosslinker, solvent and a Lewis base as a catalyst [1]. The result of the reaction was a three-dimensional rigid network with short and rigid connections. Generally, hypercrosslinked polymers are prepared by post-polymerisation crosslinking of long polymer chains in a swollen polymer matrix creating numerous new links. In the case of chloromethylated polystyrene, polymer chains are reacted with a Friedel–Crafts catalyst in a swelling solvent. This creates new connections by converting chloromethyl groups into methylene bridges that interconnect polymer chains resulting in the formation of micropores. Through further stages of the hypercrosslinking process, the crosslinking increases and, therefore, after solvent removal, a stable microporous network is formed [2,3]. The hypercrosslinked polymers may collapse during drying however reinforcement of their skeleton with additional crosslinks enables their re-expansion both with polar and non-polar solvents [4,5]. Hypercrosslinked polymers contain a very high density of crosslinks together with micropores and exhibit high surface area (up to 2000 m^2^/g) [4,6].

The main difference between a post-polymerisation hypercrosslinking and copolymerisation crosslinking is in the pore formation method. In copolymerisation crosslinking, pores are formed due to phase separation during polymerisation between the monomer and the crosslinker, in the presence of an inert diluent. The result is the formation of a two-phase heterogeneous system where one phase represents a crosslinked polymer and the other excess diluent and possibly unpolymerised monomers. Usually, phase separation occurs near the gel point and can appear as macrosyneresis [7] when the fluid and a swollen gel make two continuous phases. Resulting macroreticular polymers have a cauliflower-like morphology [8,9,10], with pores as spaces in clusters of crosslinked polymer nodules. Such polymers usually exhibit relatively modest specific surface area and somehow poorer mechanical stability [11] than their gel-type counterparts do. Post-polymerisation crosslinking—hypercrosslinking performed on swollen polymers induces the formation of additional porosity and increases rigidity of the polymer matrix which improves its compatibility with both thermodynamically good and bad solvents [5,12] and thus improve the accessibility of reactive sites. Therefore, hypercrosslinked polymers are used as separation columns [13,14,15,16], adsorbents [17,18,19,20,21], solid state supports for catalysts [22,23,24], to name the most frequent applications.

On the other hand, macroporous polymers in monolithic form can be obtained by the polymerisation of a continuous phase of high internal phase emulsion (polyHIPE) [25,26,27,28]. The internal (or droplet) phase is dispersed in the continuous phase of the emulsion and represents at least 74.05% of the total volume of the emulsion in the uniform packing of monodispersed droplets, or 64% in the case of random packing [29]. Most common are water-in-oil HIPEs, where the continuous phase consists of monomers, surfactants and initiator, while the internal phase is aqueous. After the polymerisation of the continuous phase, the droplet phase is removed, and macro pores are thus formed. The internal topology of polymeric material prepared in this way has two levels of pores, the primary pores, and secondary, interconnecting pores. PolyHIPEs have so far found numerous applications such as tissue engineering [30,31,32,33], as columns in separation systems [34,35,36,37,38], as supports for catalysts [6,39,40] etc.

Hypercrosslinking of 4-vinylbenzyl chloride (VBC)-based polyHIPEs results in rigid polymers with induced microporosity in macro porous material, which leads to very high specific surface areas and better compatibility with solvents. This makes the sites within such a hierarchically porous polymer more accessible. In flow systems, macro pores allow for reduced back pressure and faster mass transfer, while micro and meso pores lead to an increase in specific surface area and thus greater availability of reactive sites. It was demonstrated that polyHIPEs can be hypercrosslinked even without the introduction of functional monomer e.g., VBC [41]; however, a more efficient method for creating additional crosslinking is based on the Friedel–Crafts catalysed transformation of chloromethyl groups into crosslinking methylene bridges [8]. A convenient way to introduce chloromethyl groups into styrenic polymers is admixture of VBC to the mixture of co-monomers. Schwab et al. [42] prepared hypercrosslinked VBC/DVB polyHIPEs with a DVB content between 2.5 and 40 mol.%. After hypercrosslinking, the surface area increased significantly (to 1200 m^2^/g), while the polyHIPE morphology remained unchanged. The materials showed to be promising for n-butane storage. In another study VBC based polyHIPEs with 2 mol% DVB content were used for controlled hypercrosslinking to leave some unreacted benzyl chloride groups for further binding of MAP (methyl amino pyridine) [6]. Monolithic VBC polyHIPE was found to be a very effective nucleophilic catalyst. Porous carbon with a high BET specific surface area and hierarchical porous structure was synthesised by pyrolysis of hypercrosslinked VBC/DVB polyHIPE [43]. Hypercrosslinking generated micro pores to limit the degradation of polyHIPE morphology after pyrolysis.

Despite a lot of research on post-polymerisation hypercrosslinking of VBC based polymers and numerous applications (see Reference [44] for a recent review), there is a lack of information about the influence of VBC polymer chain concentration and of initial crosslinking degree on the post-polymerisation crosslinking process. Herein, we report the results of the study applying VBC based polyHIPEs with various initial crosslinking degrees in the process of a Friedel–Crafts type post-polymerisation hypercrosslinking process.

## 2. Materials and Methods

### 2.1. Materials

We purified 4-vinylbenzyl chloride (VBC, 90%, Sigma Aldrich, St. Louis, MO, USA), styrene (STY, Sigma Aldrich) and divinylbenzene (DVB, 80%, composed of 80% of divinylbenzene and 20% of ethylvinylbenzene, Sigma Aldrich) by passing them through a layer of aluminium oxide (Fischer Chemical, Guangzhou, China) to remove the inhibitors. Sorbitan monooleate (Span 80, Sigma Aldrich), α,α’-azo-bisisobutyronitrile (AIBN, Fluka, Buchs, Switzerland), acetone (Sigma Aldrich), methanol (Sigma Aldrich), 1,2-dichloroethane (DCE, 99,5%, Sigma Aldrich), nitric acid (HNO_3_, 63%, Carlo Erba, Sabadell, Spain), calcium chloride hexahydrate (CaCl_2_·6H_2_O, Sigma Aldrich) were used as received.

### 2.2. Preparation of 4-Vinylbenzyl Chloride/Divinylbenzene/Styrene Poly(High Internal Phase Emulsions) (PolyHIPEs)

An internal phase consisting of a 1.76% aqueous solution of CaCl_2_·6H_2_O was added dropwise under constant stirring to a continuous phase, consisting of 4-vinylbenzyl chloride (VBC), styrene (STY) and divinylbenzene (DVB) monomers (at different molar ratios, see Table 1), surfactant Span 80 (20 vol% with regards to monomers) and initiator AIBN (1 wt.% with regards to monomers). The emulsion was stirred for a further 60 min after the addition of the internal phase at 250 rpm and then poured into polypropylene moulds and transferred to the oven for 24 h at 60 °C to complete the polymerisation. After polymerisation, the monoliths were purified by Soxhlet extraction (24 h in deionized water and 24 h in acetone) and then air-dried. The polyHIPEs are labelled according to their DVB content (A 2 mol.%, B 5 mol.% and C 10 mol.%), and the molar percentage of VBC in the monomer mixture. Detailed information on the monomer mixture composition is given in Table 1.

### 2.3. Hypercrosslinking of PolyHIPEs

A powdered polyHIPE sample (1 g) was added to a two-necked round-bottom flask, along with 80 mL of 1,2–dichloroethane. After stirring and purging with nitrogen for 15 min, the mixture was stirred for a further 45 min to allow the sample to swell. A FeCl_3_ catalyst (relative to the calculated chloromethyl groups) was added through the neck of the flask, which was placed on an ice bath. This was followed by a 15 min nitrogen purge and then a 45 min constant stirring under nitrogen atmosphere. The flask was then placed on an oil bath at 80 °C. After 20 min, the sample was removed from the oil bath and the reaction was quenched by the addition of 40 mL of methanol. Hypercrosslinked polyHIPEs were washed with methanol and 0.1 M HNO_3_ (3 × 20 mL of each). The final products were purified by Soxhlet extraction in acetone for 24 h and air-dried.

### 2.4. Characterization

Nitrogen adsorption/desorption measurements were done on a Micromeritics TriStar II 3020 (Norcross, GA, USA) porosimeter using the manufacturer’s software.

SEM pictures were taken on a Phillips XL-30 SEM (Leuven, Belgium) operating at 20 kV. Samples were platinum coated using ion sputter Jeol JCF-1100E (Tokyo, Japan) for 2 min at 40 mA.

Chlorine content was determined as follows: after homogenization of the samples, a small amount of the sample, typically 1 mg, was weighed into a sillica holder and transferred to an AOX combustion tube. The samples were combusted using a Haberkorn/Braun M 2000C instrument (Dietikon, Switzerland) in an oxygen atmosphere at 950 °C, converting the chlorine to chloride ions. The final amount of chloride was determined by an automated coulometric titration. Analyses were performed in triplicate.

## 3. Results

### 3.1. Preparation of Poly(VBC-co-DVB-co-STY)HIPEs

**Figure 1:**
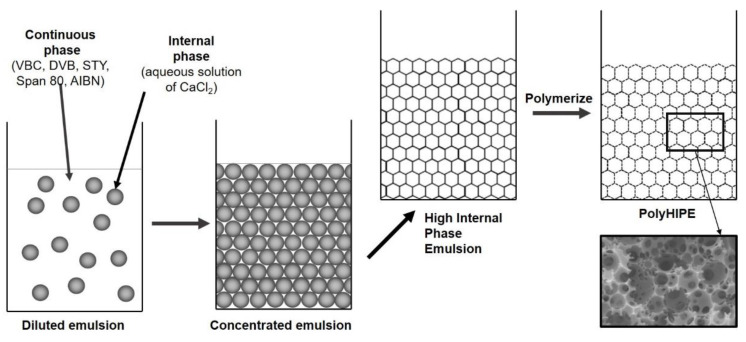
PolyHIPE preparation.

Poly(VBC-co-DVB-co-STY)HIPEs were prepared with varied ratios of VBC, DVB, and STY monomers (Figure 1). Using free radical polymerisation initiated by AIBN, polyHIPE monoliths with three different crosslinking degrees were synthesized. Firstly, the influence of initial DVB-caused crosslinking, on the degree of hypercrosslinking, was evaluated. For this purpose, 2, 5 and 10 mol.% of DVB were used as initial crosslinking degree. Rather low degrees of initial crosslinking was used as it was expected that the rigidity of the polymer network would have a substantial diminishing effect on the post polymerisation hypercrosslinking process. Most examples from the literature describe a Friedel–Crafts type of hypercrosslinking performed on poly(4-vinylbenzyl chloride) with a low degree of crosslinking, up to 5 mol.% [4,6,45,46], while much less is known on this type of hypercrosslinking of networks with higher degree of crosslinking. Furthermore, we were interested in how the concentration of the hypercrosslinking monomer, in our case 4-vinylbenzyl chloride, influences the hypercrosslinking process. As the process involves a reaction between the neighboring functional groups, it is expected that the distribution of functional groups within the polymer network would have a significant effect on the formation of crosslinks during the hypercrosslinking process. Therefore, the experimental design included variation of both initial crosslinking degree (DVB content) and “dilution” of functional groups i.e., the ratio of functional to “diluting” monomer (styrene was used for this purpose). It was assumed that all added DVB to the monomer mixture was crosslinking as the highest addition of DVB was 10 mol.%. Three series of experiments were performed, one for each initial crosslinking degree (Table 1). In addition to DVB, STY was added to each batch (from 5 to 65 mol.% of monomers, Table 1), and the rest was VBC. Chloromethyl functional groups in the monomeric system was diluted with the addition of STY to obtain different VBC contents at the same initial crosslinking degrees, which proved to be an important factor in further hypercrosslinking studies of synthesized polyHIPEs.

The kinetic stability of the emulsion is extremely important for the formation of polyHIPE monoliths and was achieved with the addition of 20 wt.% of the surfactant Span 80 (HLB = 4.3) from the oleate family of surfactants, which is suitable for reducing the interfacial tension in w/o emulsions. With the addition of surfactant, stable HIPEs and polyHIPEs were obtained with typical interconnecting open cell polyHIPE morphology and average diameter of primary pores between 5 and 10 µm.

### 3.2. Hypercrosslinking of PolyHIPEs

Hypercrosslinking of polyHIPEs was performed by an established procedure, applying a Friedel–Crafts type electrophilic aromatic substitution process [6]. Generally, a chloromethyl group tethered to the polymer chain can react either with a benzene ring or another VBC moiety creating methylene bridges or cyclical type connections. DCE has been used as a solvent in which styrene-type polymers are swollen, while being effective in dissolving FeCl_3_, which serves as a catalyst for Friedel–Crafts alkylation reactions. The effect of this procedure was apparent both in the drop of chlorine content due to conversion of a part of chloromethyl groups into crosslinking methylene bridges and in a substantial increase of the BET surface area (Table 2).

**Figure 2:**
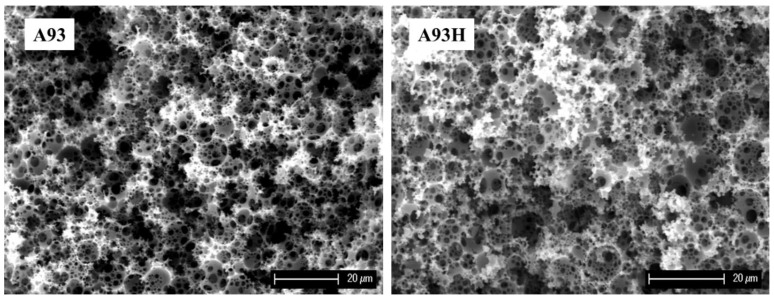
Scanning electron micrographs of polyHIPE samples A93 and A93H.

In most cases, the residual chlorine content after hypercrosslinking was 10–15 wt.%. Somewhat surprisingly, higher values of residual chlorine (suggesting lower efficiency of the hypercrosslinking) were found in samples with the highest VBC or DVB content. In these samples, there is greater probability that within the polymer network some chloromethyl groups could be located in less accessible places. A very profound effect of the hypercrosslinking is evident in the surface area values. Before hypercrosslinking all the polymers exhibited low surface area only, corresponding with the area of the macro pores created by emulsification of the monomer mixture. The hypercrosslinking porosity modification does not modify the macroporous polyHIPE features (Figure 2).

Substantially higher BET surface of the hypercrosslinked polymers indicates the formation of new porosity. However, hypercrosslinking is known to produce microporosity with pores smaller than 2 nm [47]. Such narrow pores are filled with the sorbate sooner than the BET equation-described multilayer adsorption begins. Standard evaluation of the BET surface of such materials interprets the sorbate amount filling the micropores as a part of the BET multilayer, which produces incorrect BET surface area data. It is possible to distinguish between micropore volume and external surface area using the *t*-plot method, which is plotting the adsorbed amount against thickness of the adsorbed layer computed from adsorption on a flat surface. The software used in this study is based on the Harkins and Jura thickness equation [48]. The linear part of such a plot (usually corresponding to relative pressures 0.05–0.35) can be considered as the region of the adsorption on flat surface. The Y-intercept of such a line corresponds to the micropore volume and the slope is proportional to the external (outside micropores) surface area [49].

**Figure 3:**
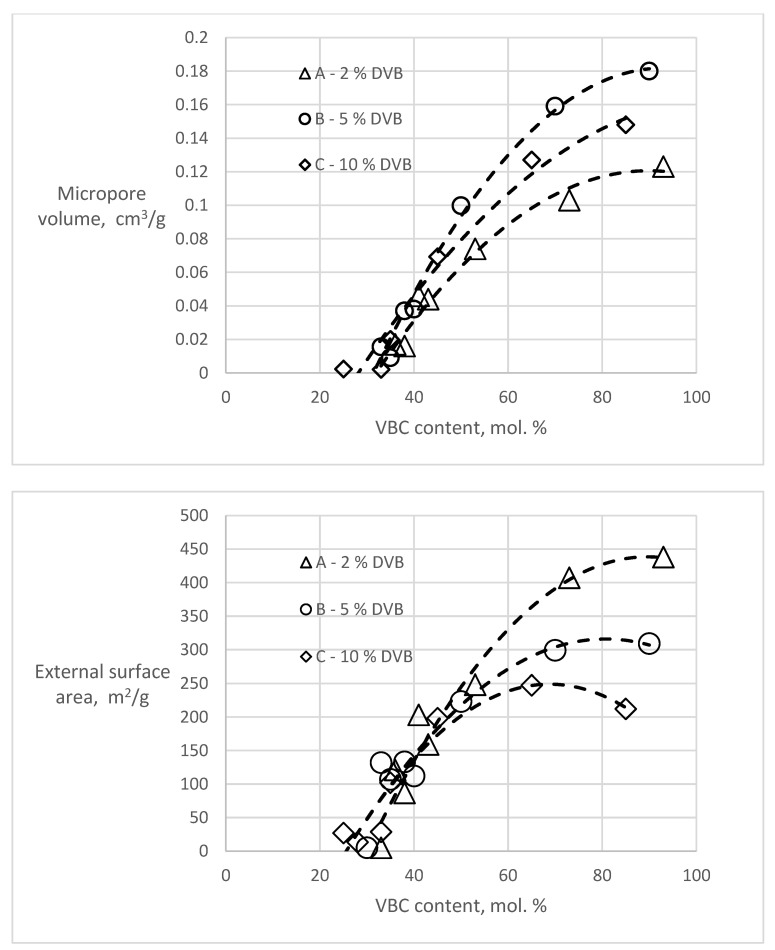
Dependencies of micropore volume and external surface area in the hypercrosslinked polymers on the content of VBC.

T-plot analyses of the polymers before hypercrosslinking show zero or slightly negative values of the micropore volumes (−0.008–0 cm^3^/g) and external surface areas in the range of 0.8–8.8 m^2^/g, slightly higher than the BET surfaces. However, considering the completely different theoretical background of these two methods, the agreement is very good. In the hypercrosslinked polymers, both the amount of micropores and the external surface area varied in dependence of the VBC content of the polymer (Figure 3).

Both dependencies show that for the formation of new porosity, the presence of chloromethyl groups in about one third of the monomer units is needed. With increasing VBC concentration, both the micropore volume and the external (mesopore) surface area increase. The character of the newly created porosity and the effect of the DVB content in the starting polymers is most apparent in the samples A93, B90 and C85 having the highest VBC content in the series (Table 3). It is evident that with an increase of the DVB content (crosslinking) of the starting polymer the total volume of the hypercrosslinking-generated pore volume decreases. An increase of the crosslinking of the starting polymer also influences the pore size distribution. While with 2 mol.% DVB in the starting polymer the micropores formed in the hypercrosslinked material represent only about one quarter of the newly formed porosity, in the hypercrosslinked polymer C85 containing 10 mol.% DVB they represent half of the total porosity.

## 4. Conclusions

Concentration of functional groups has been shown to have a significant effect on the hypercrosslinking process of vinylbenzyl chloride-based polyHIPEs. The strong influence of VBC concentration is evident at all the three initial crosslinking degrees. The VBC content of about 30 mol.% appears to be a critical value above which starts the formation of new pores having dimensions from micropore (<2 nm) to mesopore (tens of nm). Pore formation during hypercrosslinking proceeds within swollen polymer gel by a gradual increase of the local polymer chain density probably inducing phase separation in the form of liquid droplets within a continuous polymer matrix that is by microsyneresis. Hence, the more swollen the starting polymer is (the lower is its DVB content) the greater the volume of new pores can be created. The level of the DVB content in the starting polymer also influences the pore size distribution of the created pores which is evident in the increase of the share of micropores with the increase of the DVB content. The results shed more light on the Friedel–Crafts hypercrosslinking process in polyHIPEs, allowing for more control of the microstructure and the preparation of polymer materials with controlled morphology on several porosity levels.

## Figures and Tables

**Table 1:** Composition of poly (4-vinylbenzyl chloride (VBC)/divinylbenzene (DVB)/ styrene (STY)) poly(high internal phase emulsions) (polyHIPE) samples.

Sample	Continuous Phase			V_AP_ * [mL]	m (Span 80) [g]
	m (STY) [g]	m (DVB) [g]	m (VBC) [g]		
A93	0.181	0.095	4.747	18.6	1.264
A73	0.899	0.086	3.724	17.9	1.185
A53	1.563	0.107	2.696	17.2	1.162
A43	1.909	0.106	2.225	16.9	1.035
A41	1.98	0.091	2.104	16.8	0.83
A38	2.091	0.111	1.936	16.8	0.834
A36	2.111	0.450	1.539	16.7	0.887
A33	2.253	0.087	1.679	16.5	1.005
B90	0.173	0.257	4.579	18.6	1.339
B70	0.877	0.218	3.567	17.9	1.199
B50	1.583	0.234	2.573	17.2	1.089
B40	1.926	0.258	2.035	16.9	1.076
B38	1.98	0.217	1.933	16.8	0.916
B35	2.096	0.246	1.794	16.7	0.817
B33	2.187	0.226	1.630	16.6	0.815
B30	2.279	0.221	1.532	16.5	1.035
C85	0.188	0.440	4.372	18.6	1.227
C65	0.566	0.498	3.858	18.3	1.194
C45	1.223	0.457	2.814	17.6	1.122
C35	1.922	0.443	1.788	16.9	1.087
C33	1.978	0.438	1.678	16.8	0.835
C28	2.196	0.458	1.386	16.6	0.814
C25	2.256	0.460	1.290	16.4	0.993

* Volume of the aqueous phase. Samples A:2 mol.% DVB, B: 5 mol.% DVB, C:10 mol.% DVB.

**Table 2:** Hypercrosslinking effects.

Sample	Before Hypercrosslinking		After Hypercrosslinking		
	Cl Content, wt.%	BET Surf. Area, m^2^/g	Cl Content, wt.%	Residual Cl, % of Orig.	BET Surf. Area, m^2^/g
A93	21.90 ± 0.20	2.40	3.70 ± 0.03	16.9	709
A73	18.00 ± 0.40	3.20	1.70 ± 0.03	9.4	636
A53	13.90 ± 0.20	4.60	1.80 ± 0.10	12.9	410
A43	11.70 ± 0.10	1.80	1.00 ± 0.02	8.5	279
A41	11.20 ± 0.30	2.30	2.00 ± 0.10	17.9	304
A38	10.30 ± 0.30	3.00	1.40 ± 0.20	13.6	122
A36	8.20 ± 0.10	3.10	1.30 ± 0.10	15.9	5.0
A33	9.20 ± 0.20	3.90	0.60 ± 0.03	6.5	3.9
B90	21.30 ± 0.60	4.40	7.50 ± 0.40	35.2	585
B70	17.80 ± 0.40	6.00	2.70 ± 0.10	15.2	491
B50	13.70 ± 0.20	4.80	1.70 ± 0.04	12.4	412
B40	11.00 ± 0.30	2.30	1.10 ± 0.10	10.0	196
B38	10.40 ± 0.10	3.10	1.20 ± 0.10	11.5	217
B35	9.60 ± 0.10	2.30	1.40 ± 0.10	14.6	70
B33	8.90 ± 0.50	3.40	1.10 ± 0.10	12.4	8.0
B30	8.30 ± 0.20	3.00	0.80 ± 0.10	9.6	4.2
C85	20.40 ± 0.50	2.20	4.10 ± 0.03	20.1	536
C65	18.20 ± 0.30	3.80	2.30 ± 0.10	12.6	547
C45	14.30 ± 0.20	3.60	1.50 ± 0.07	10.5	350
C35	9.60 ±	2.10	1.20 ± 0.03	12.5	62
C33	9.20 ± 0.10	2.60	1.40 ± 0.10	15.2	14
C28	7.60 ± 0.50	3.10	0.80 ± 0.10	10.5	14
C25	7.10 ± 0.40	1.80	1.00 ± 0.10	14.1	13

Samples A: 2 mol.% DVB, B: 5 mol.% DVB, C:10 mol.% DVB.

**Table 3:** Comparison of micro- and meso-porosity created by hypercrosslinking.

Sample	V_p_ [cm^3^/g]	V_micro_ [cm^3^/g]	V_meso_ [cm^3^/g]	V_micro_/V_p_
A93 (2 mol.% DVB)	0.43	0.12	0.31	0.28
B90 (5 mol.% DVB)	0.40	0.18	0.22	0.45
C85 (10 mol.% DVB)	0.30	0.15	0.15	0.50

## Data Availability

Not applicable.
